# Molecular cloning and expression patterns of the cholesterol side chain cleavage enzyme (CYP11A1) gene during the reproductive cycle in goose (*Anas cygnoides*)

**DOI:** 10.1186/s40104-015-0053-9

**Published:** 2015-12-22

**Authors:** Qi Xu, Yadong Song, Yang Chen, Ran Liu, Yang Zhang, Yang Li, Zhengyang Huang, Wenming Zhao, Guobin Chang, Guohong Chen

**Affiliations:** Key Laboratory of Animal Genetics and Breeding and Molecular Design of Jiangsu Province, Yangzhou University, Yangzhou, 225009 PR China

**Keywords:** *CYP11A1*, Gene expression, Goose, Reproduction

## Abstract

**Background:**

*CYP11A1*, a gene belonging to the family 11 of cytochrome P450, encodes a crucial steroidogenic enzyme that catalyzes the initial step in the production of all classes of steroids. Many studies show that *CYP11A1* plays a role in ovary function. However, the role of *CYP11A1* in goose reproductive cycle remains largely unknown.

**Results:**

In this study, full-length *CYP11A1* cDNA of Zhedong goose was obtained using reverse transcription polymerase chain reaction (RT-PCR) and rapid amplification of cDNA ends (RACE). The cDNA consisted of a 96-base pair (bp) 5′untranslated region (UTR), a 179-bp 3′UTR and a 1509-bp open reading frame. The open reading frame encodes a putative 503 amino acid protein that shares high homology with CYP11A1 of other birds. The amino acid sequence possesses conserved domains of the P450 superfamily, which include the steroid-binding domain and the heme-binding region. Real-time quantitative polymerase chain reaction (qPCR) analysis revealed *CYP11A1* mRNA was expressed ubiquitously in every Zhedong goose tissue analyzed, including the heart, liver, glandular stomach, lung, spleen, kidney, intestinum tenue, intestinum crassum, cerebrum, cerebellum, muscle, oviduct, pituitary, hypothalamus and ovary.. The relatively low levels of *CYP11A1* mRNA were detected in pituitary, ovary and oviduct tissues at ovulation when compared with levels at oviposition. Interestingly, higher expression was observed in ovary and oviduct tissues during brooding. Lastly, higher mRNA expression of Yangzhou geese was detected during the ovulation period than that of Zhedong geese.

**Conclusions:**

Our findings reveal the sequence characterization and expression patterns of the CYP11A1 gene during the goose reproductive cycle, which may provides correlative evidence that CYP11A1 expression is important in reproduction activity.

## Background

Cytochrome P450 side chain cleavage (P450scc, encoded by the *CYP11A1* gene) plays a major role in the regulation of steroidogenesis by mediating the conversion of cholesterol to pregnenolone [1]. Because of its physiological importance, *CYP11A1* has been the focus of many studies. Recently, more and more evidence shows that CYP11A1 plays a role in ovary function. In fowl, many investigations have found that *CYP11A1* expression changes dynamically in developing follicles [2–4] and that its expression in ovarian granulosa cells is influenced largely by hormonal regulation [5–9]. Another study found that the hormonally and developmentally regulated expression of *CYP11A1* was principally driven by multiple trans-acting factors, like Sp-1 [10, 11], SF-1/LRH-1, GATA4, CREB-1,AP-1 [12] AP-2, LBP-1b/LBP-9 [13] and Ff1b [14].

The goose (*Anas cygnoides*) is a commercially important food source that is widely cultivated in China. It is an ideal animal model for characterization of fowl reproduction because of obvious reproductive stages and strong broodiness [15]. In a previous study, we identified *CYP11A1* as an important candidate gene through transcriptome profiling of ovaries from laying and brooding geese [16]. To further characterize the role of *CYP11A1* in the goose reproductive cycle, we cloned Zhedong goose (*Anas cygnoides*) *CYP11A1* and characterized its spatiotemporal expression patterns by Real-time qPCR. We also investigated the differences in *CYP11A1* expression in high egg production breed(Yangzhou geese, *Anas cygnoides*) and low egg production breed(Zhedong goose). These data may facilitate a better understanding of expression patterns of the CYP11A1 gene during the reproductive cycle in goose.

## Methods

### Ethics statement

All animal experiments were reviewed and approved by the Institutional Animal Care and Use Committee of Yangzhou University. Procedures were performed in accordance with the Regulations for the Administration of Affairs Concerning Experimental Animals (Yangzhou University, China, 2012) and the Standards for the Administration of Experimental Practices (Jiangsu, China, 2008).

### Animals and tissue sample collection

One hundred female Zhedong geese and Yangzhou geese were selected from the breeding farm of Jiangsu Lihua Animal Husbandry Co. Ltd (Changzhou, China)), which laid about thirty and seventy eggs, respectively. They were both housed semi-enclosed building and raised the same conditions according to the farm’s standard practice. During the experiment, geese were fed ad libitum with rice grain supplemented with green grass or water plants whenever possible. The feed was provided during the daytime when the geese were released to an open area. The geese were exposed to natural light and ambient temperature throughout this study. The five 120-day-old young (prelaying) Zhedong female geese were sacrificed to investigate *CYP11A1* expression patterns in different tissues. The Zhedong geese were anesthetized with sodium pentobarbital and the heart, liver, glandular stomach, lung, spleen, kidney, intestinum tenue, intestinum crissum, cerebrum, cerebellum, muscle, oviduct(infundibulum), pituitary, hypothalamus and ovary(stroma) were removed and immediately frozen in liquid nitrogen before storage at−80 °C for RNA isolation. Tissue samples from the oviduct(infundibulum), pituitary gland, hypothalamus, and ovary(stroma) were obtained from twelve sacrificed 380-day-old adult geese, including three laying Zhedong geese with an egg in the oviduct (ovulation, the release of an ovum from a ruptured follicle), three laying Zhedong geese without an egg in the oviduct (oviposition, the laying of the egg), three brooding Zhedong geese, and three laying Yangzhou geese with an egg in the oviduct (ovulation). The tissue samples were obtained promptly, as described above, to characterize developmental expression patterns.

### Zhedong goose *CYP11A1* cDNA cloning and sequencing

Total RNA was extracted from collected tissue samples using TRIzol reagent according to the manufacturer’s instruction (TaKaRa, China). The RNA was resuspended in RNase-free water, and the concentration and purity were determined using a NanoDrop Spectrophotometer (NanoDrop, USA). After purification, total RNA (2 μg) was reverse transcribed using M-MLV reverse transcriptase (Promega, USA) according to the manufacturer’s protocol. BLAST analysis of one unigene revealed that it was highly similar to the chicken *CYP11A1* gene [17]. Primers were designed according the unigene (Table 1) and RT-PCR was performed using ovarian cDNA from geese. The PCR product was purified, cloned into the pMD19-T vector (TaKaRa, China), and subjected to sequence analysis. The 5′ and 3′ ends of *CYP11A1* were amplified via rapid amplification of cDNA ends (RACE) using the SMART RACE cDNA amplification protocol (Clontech, USA) and the 3′-Full RACE Kit (TaKaRa, China), respectively. RACE primers (Table 1) were designed using the partial *CYP11A1* nucleotide sequence obtained from RT-PCR. Touchdown and nested PCRs were performed according to the manufacturer’s instructions. Amplicons were then cloned into a plasmid vector for nucleotide sequencing by Sangon Biotech (Shanghai, China).

**Table 1:** Primers used for gene cloning and expression analysis

Primer name	Oligo sequences (5′–3′)	Type
cCYPF	GTCTGTGTGCCATGTGCTGTACGG	Complete ORF
cCYPR	GTTTGTCGGGGAGGAGGATGAGGT	
5′Router	GAACTTGCGGGCCATGATGT	5′RACE
5′Rinner	CCCGCAGCCGGACGACC	
3′Router	CAAGCACTTCAAGGGGCTGAGCTT	3′RACE
3′Rinner	GACCAAGCGGGCAGTGGAAGTTGGGACCA	
eCYP-F	TGCTGCAGGACTTTGTGG	Expression profile
eCYP-R	TGGAGAGGATGCCCATGT	
GAPDH-F	GGTGGTGCTAAGCGTGTCAT	Expression profile
GAPDH-R	CCCTCCACAATGCCAAAGTT	

### Sequence analyses

The Zhedong goose CYP11A1 cDNA and the deduced amino acid sequences were analyzed using DNAssist (version 2.2) and the Expasy search program (http://au.expasy.org/tools/), respectively. Homology analyses were carried out using Clustal W (http://www.ebi.ac.uk/Tools/msa/). The phylogenetic analyses and statistical neighbor-joining bootstrap tests of the phylogenies were performed using the MEGA package (version 6.0) and the bootstrap method was used to indicate confidence values for tree nodes based on 1000 replicates. The conserved domain analysis was predicted by SMART (http://smart.embl-heidelberg.de/).

### *CYP11A1* mRNA expression patterns in Zhedong goose

To study *CYP11A1* mRNA expression, we performed Real-time qPCR on total RNA isolated from the tissues of prelaying Zhedong geese and the kidney tissue served as a calibrator tissue. Assays were conducted in 20 μL reactions using the SYBR Premix Ex Taq^TM^ (TaKaRa, China) and performed on an ABI two-step RT-PCR system (Applied Biosystems 7500, USA) with diluted first-strand cDNA. qPCR programs for *CYP11A1* and glyceraldehyde-3-phosphate dehydrogenase (*GAPDH*) were: 1 cycle of 95 °C for 5 min,40 cycles of 95 °C for 10 s, 60 °C for 34 s data collection, followed by 1 cycle for melting curve analysis. All cDNA synthesis reactions were carried out using 100 ng of total RNA per reaction and assayed in three to four technical replicates for each set of biological samples. The same methods were used to determine the Zhedong *CYP11A1* mRNA expression profile during the reproductive cycle. The *GAPDH* gene served as an internal reference gene and the hypothalamus tissue from prelaying Zhedong geese served as a calibrator tissue for differential expression analyses. To analyze the difference of expression patterns between different egg production goose breeds(Zhedong geese and Yangzhou geese), the mean ΔCt value of the hypothalamus tissue of Zhedong geese within each group was used as the calibrator.

### Statistical analyses

Statistical analyses were performed using t-tests independent of group and SPSS version 13.0 software.

## Results

### Zhedong goose CYP11A1 cDNA cloning and sequence analyses

To obtain the full-length cDNA of goose *CYP11A1*, RT-PCR and RACE were carried out. The *CYP11A1* cDNA was only found to be 1784 nucleotides in length, which included a 96-nucleotide 5′ UTR, a 179-nucleotide 3′ UTR, and a 1509-nucleotide open reading frame putatively encoding a single 503 amino acid protein. The other transcript variant did not been found among the tissues tested in Zhedong goose.

### Phylogenetic analysis of the putative CYP11A1

**Fig. 1:**
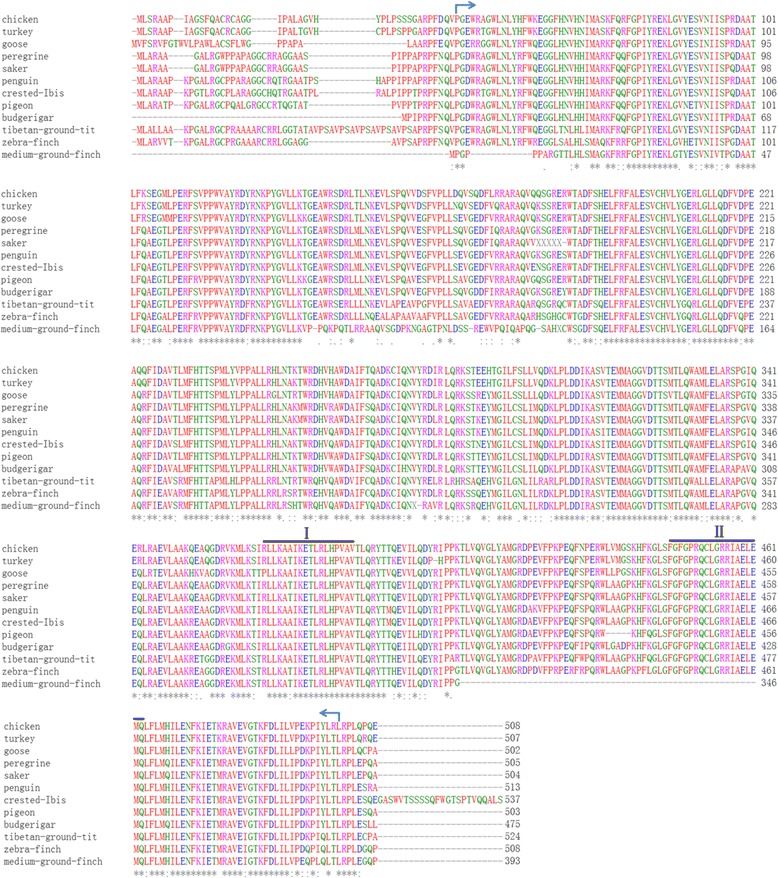
Alignment of the deduced amino acid sequence of goose CYP11A1 with those of the other birds species. Additional Genbank accession numbers not mentioned elsewhere are as follows: chicken(*Gallus gallus*,NM_001001756.1), turkey(*Meleagris gallopavo*,XR_118355.1), penguin (*Aptenodytes forsteri*,XM_009278905.1), crested ibis(*Nipponia Nippon*, XM_009469738.1), peregrine(*Falco peregrinus*, XM_005239431.1), rock pigeon(*Columba livia*, XM_005513042.1), budgerigar(*Melopsittacus undulates*,XM_005145776.1), saker(*Falco cherrug*,XM_005437500.1), Tibetan ground-tit (*Pseudopodoces humilis*, XM_005521726.1), Zebra Finch(*Taeniopygia guttata*, NM_001127374.1) and Medium ground finch(*Geospiza fortis,* XM_005430050.1) The asterisk indicates residues that are identical among all birds. Dashes indicate gaps introduced to facilitate alignment. Arrows highlight the p450 domain, the steroid-binding domain (I), and the heme-binding region (II)

To evaluate the relationship between goose CYP11A1 and that of other birds, sequence alignment and phylogenetic analysis were carried out. Alignment of the amino acid residues of goose CYP11A1 with those of other birds forms is shown in Fig. 1. Goose CYP11A1 shared fairly high identity with birds orthologs (over 69 % identity), including budgerigar (86.3 % identity), chicken (85.6 % identity), penguin (85.2 % identity), turkey (84.8 % identity), rock pigeon (84.8 % identity), peregrine (84.7 % identity), crested ibis (84.4 % identity), peregrine (83.7 % identity), Tibetan ground-tit (77.0 % identity), Zebra Finch (76.8 % identity), and Medium ground finch (69.7 % identity). Furthermore, the p450 domain (aa 38–495 in geese) was conserved in all birds sequences analyzed.

**Fig. 2:**
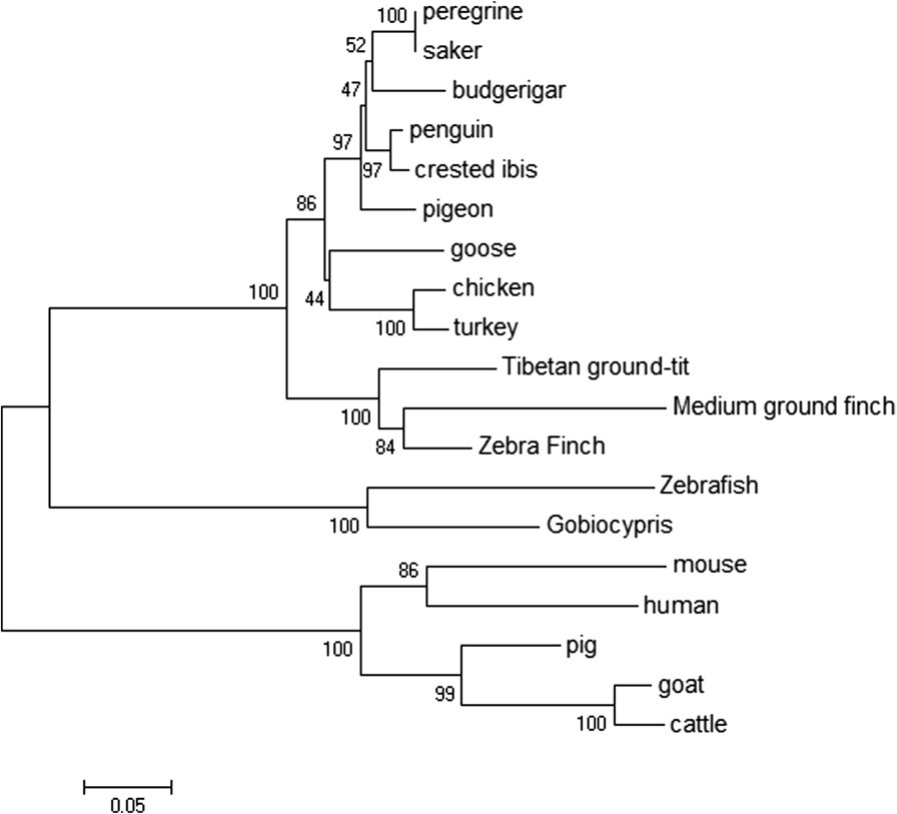
Phylogenetic analysis of CYP11A1 proteins. Phylogenetic tree constructed based on ClustalW alignment of the deduced amino acid sequences of CYP11A1 from different birds. The reliability of the neighbor-joining tree was estimated by bootstrap analysis with 1,000 replicates. Bootstrap values are shown on the lineages of the tree and major taxonomic clusters are indicated separately. Additional Genbank accession numbers not mentioned elsewhere are as follows: mouse(*Mus musculus*, NM_019779.3), zebrafish (*Danio rerio,* AF527755.1), gobiocypris(*Gobiocypris rarus,* JN858106), human(*Homo sapiens*,NM_000781), pig(*Sus scrofa*,NM_214427), goat(*Ovis aries*,NM_001093789) and cattle(*Bos Taurus*, NM_176644). The scale bar indicates 5 % amino acid divergence in sequence

Phylogenetic analysis of CYP11A1 resulted in clear segregation into three groups, one branch containing mammalian CYP11A1, one branch of birds CYP11A1, another branch of fish CYP11A1(Fig. 2). Goose CYP11A1 clustered with other Phasianidae forms and appears to be significantly diverse from Paridae forms.

### Expression pattern of *CYP11A1* in different tissues and reproductive cycle stages in Zhedong goose

**Fig. 3:**
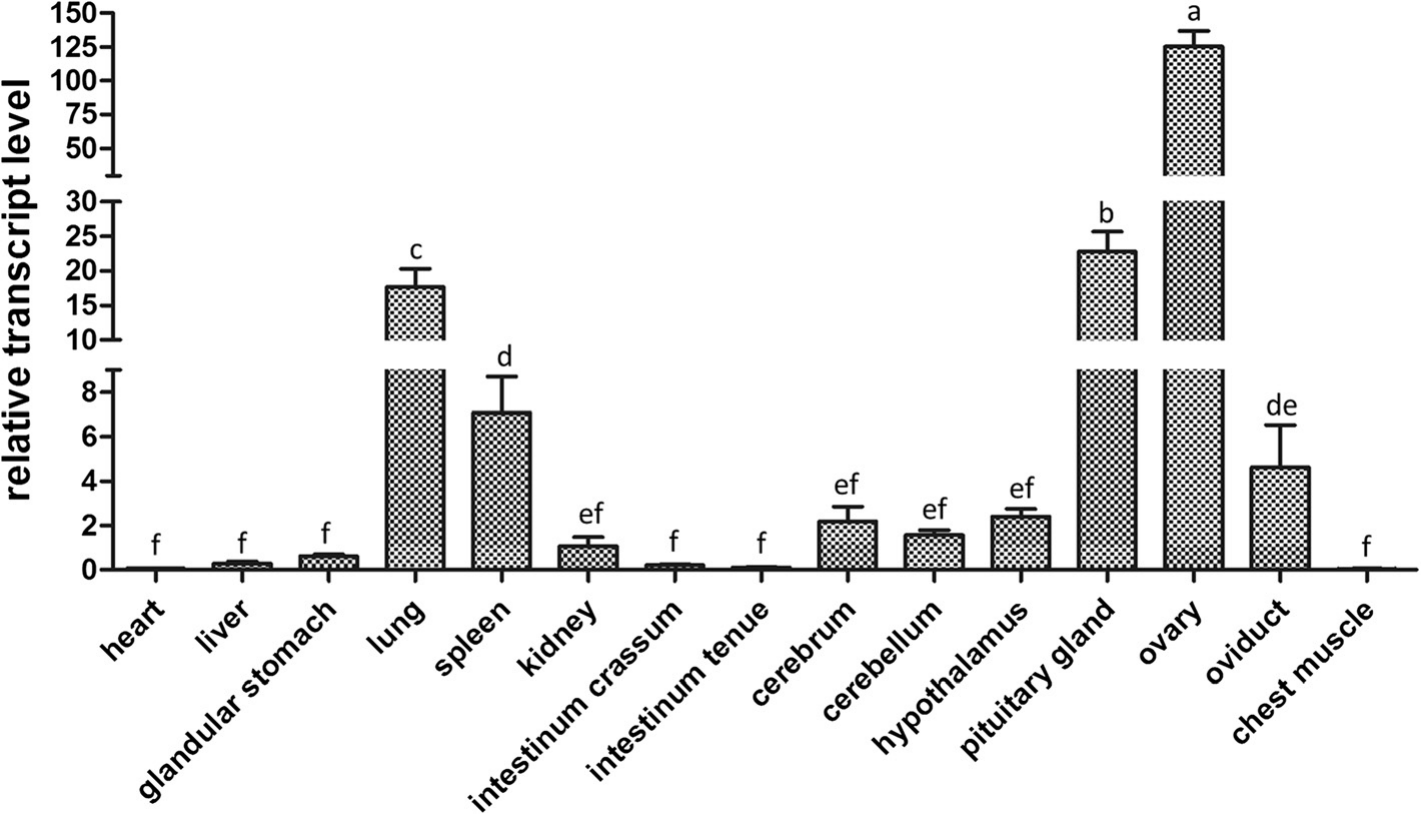
Expression of *CYP11A1* transcripts in various tissues. Gene expression was determined using Real-time qPCR and is represented relative to *GAPDH*. The tissues analyzed include the heart, liver, glandular stomach, lung, spleen, kidney, intestinum tenue, intestinum crassum, cerebrum, cerebellum, muscle, oviduct, pituitary, hypothalamus and ovary. The different lowercase letters above bars indicate significant differences between respective means (*P* < 0.05), while the same lowercase letters indicate no significant differences (*P* > 0.05)

The qPCR demonstrated that *CYP11A1* was ubiquitously expressed in fifteen tissues tested, but the expression levels were distinctly different (Fig. 3). High levels of *CYP11A1* transcript were detected in ovary, oviduct, pituitary, hypothalamus, lung, and spleen tissues in Zhedong goose, and lower negligible expression levels were found in heart and muscle tissues (Fig. 3).

**Fig. 4:**
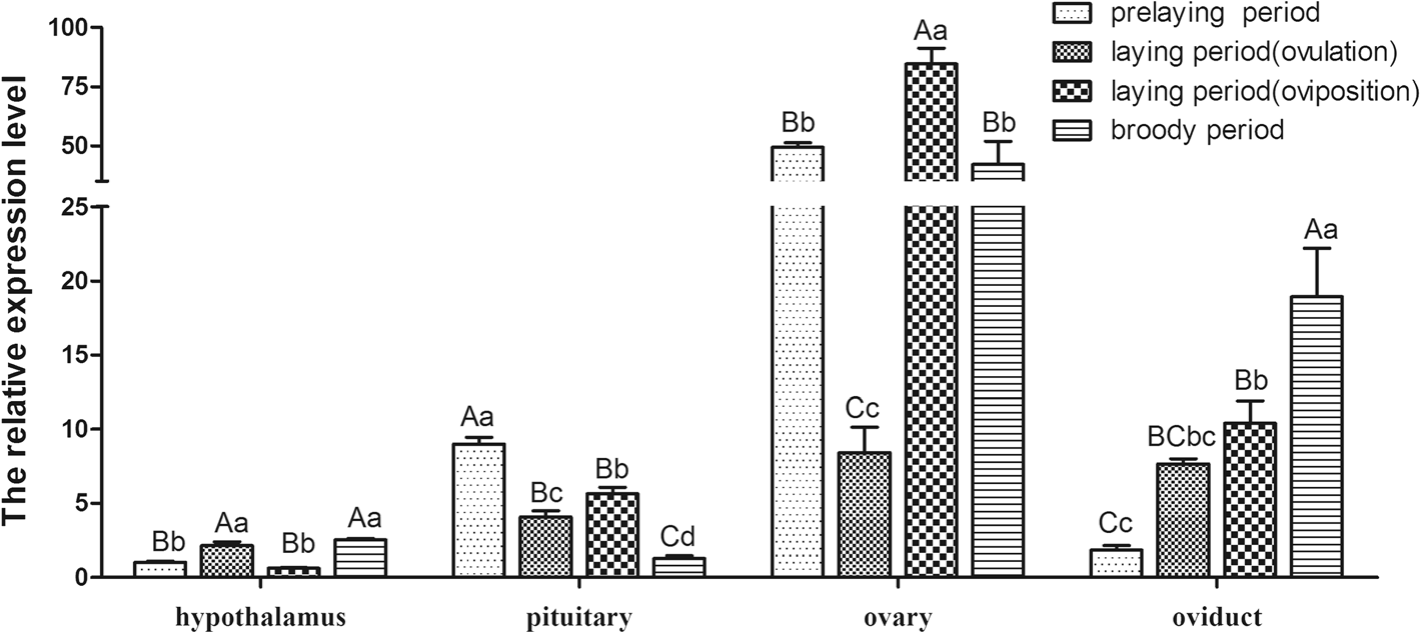
Expression patterns of *CYP11A1* during the reproductive cycle. The qPCR quantification of *CYP11A1* transcripts during the prelaying, ovulation, oviposition, and broody periods. *CYP11A1* expression was normalized to *GAPDH*. Different capital letters above bars indicate very significant differences between respective means (*P* < 0.01), while different lower case letters indicate significant differences (*P* < 0.05)

To furtherly determine temporal expression patterns we characterized *CYP11A1* expression in the ovary, oviduct, pituitary, and hypothalamus tissues during different stages of the goose reproductive cycle (prelaying, ovulation, oviposition, and broody periods). We observed different *CYP11A1* expression profiles in the different tissues at different times of the reproductive cycle (Figs. 4). From an overall perspective, it was relatively low during the ovulation period (Fig. 4). In the pituitary tissue, *CYP11A1* mRNA levels are relatively high during the prelaying period, and steadily decline through the egg laying period. In the ovary, *CYP11A1* mRNA levels dropped to an approximate tenfold decrease during the ovulation period followed by a significant increase during the oviposition period. In the oviduct, *CYP11A1* mRNA levels were relatively high during the broody period and relatively low during the prelaying period. In the hypothalamus, *CYP11A1* mRNA levels remained low throughout the entire reproductive cycle.

### Comparison on *CYP11A1* tissue-differential expression between Zhedong geese and Yangzhou geese in ovulation period

**Fig. 5:**
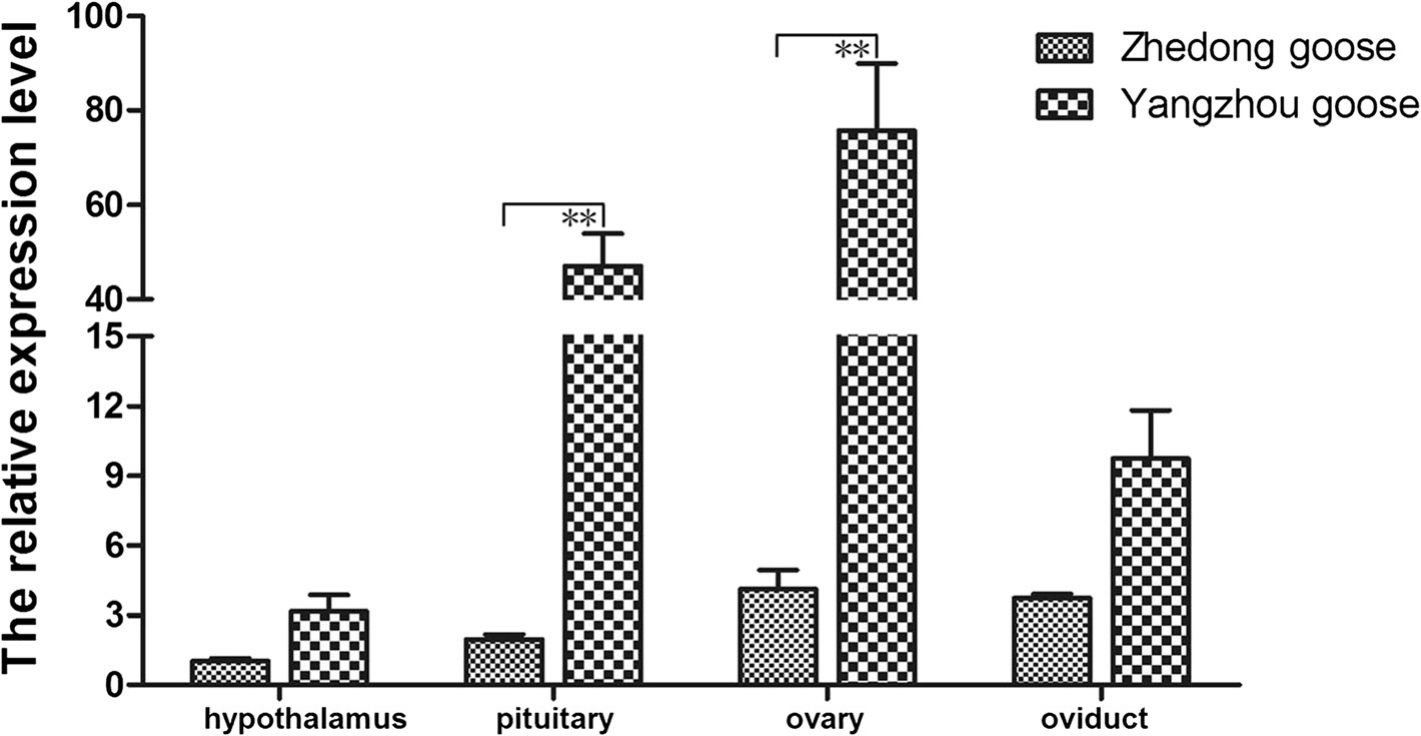
Comparison of *CYP11A1* mRNA expression between Zhedong geese and Yangzhou geese in ovulation period. Gene expression was determined using qPCR and is represented relative to *GAPDH*. Significant differences relative to controls are indicated with an asterisk (*P* < 0.01)

Additionally, to explore the differences in *CYP11A1* expression in different goose breeds, its expression levels were compared in Zhedong geese (low egg production) and Yangzhou geese (high egg production) in ovulation periods . The expression of *CYP11A* was significantly higher in the examined reproductive tissues of Yangzhou geese in the ovulation period in pituitary and ovary tissues than that of Zhedong geese (Fig. 5).

## Discussion

In this study, cDNAs encoding *CYP11A1* were cloned from Chinese goose ovaries. The deduced amino acid sequence of CYP11A1 shows high identity with that of other birds and contains some structural domains typical of the cytochrome P450 superfamily, specifically a steroid-binding domain and a heme-binding domain [18]. Both domains appear to be well conserved among the different species, indicating that they may be critical for proper enzymatic activity of all P450scc.

As expected on the basis of the predicted goose CYP11A1 amino acid sequence, phylogenetic analysis clearly clustered goose CYP11A1 with other Phasianidae forms and showed that birds CYP11A1 appear to be significantly diverged from mammalian forms and fish forms.

*CYP11A1* mRNA was expressed ubiquitously in every tissue analyzed except heart and muscle, albeit to different degrees. As expected, *CYP11A1* mRNA was easily detected in reproductive tissues and endocrine tissues. These results are in agreement with recent molecular studies aimed at determining the temporal expression of *CYP11A1* in mammals [19, 20]. The expression of the *CYP11A1* was previously well documented in tissues such as the adrenals, gonads, placenta [21, 22], pancreas [23], skin [24], gut [25], kidney [26] and certain areas of the brain [10], where its expression has been linked to steroidogenic activity. The relative abundance of specific transcripts at these various sites suggests that CYP11A1 could potentially play an important role in regulating local steroid hormone synthesis [27, 28]. We also found *CYP11A1* to be expressed in various nonendocrine tissues, including the lung, spleen, stomach, liver, brain, gut and kidney. During the last few years, *CYP11A1* has been shown to be expressed in various tissues that are not involved in steroid synthesis. Sigel A et al. have elaborated CYP11A1 own ubiquitous roles [29].

The qPCR analysis revealed that *CYP11A1* transcript abundance drastically changed in ovary, oviduct, and pituitary tissues during the different stages of the reproductive cycle. As expected, *CYP11A1* mRNA levels were relatively low at ovulation compared with oviposition. CYP11A1 catalyzes the conversion of cholesterol to pregnenolone in the first step of steroid biosynthesis [30]. It has been reported in geese and hens that the levels of progesterone in the plasma peak 2 to 3 h before ovulation and then decrease [30, 31] which might be due to low *CYP11A1* mRNA level. The higher *CYP11A1* expression was observed in ovary and oviduct tissues during broodiness, which is in concordance with previous studies that showed that gonadal steroid levels are maintained at a high level in broody birds [32]. Interestingly, The *CYP11A1* expression was high in pituitary than the oviduct during prelaying time. In contrast, the expression of gene was high in ovary/oviduct during oviposition, ovulation and brooding, but low in hypothalamus/pituitary (Fig. 4). There may be a feedback mechanism to control this gene/enzyme expression. CYP11A1 is an important enzyme to produce the progestin precursor required for the synthesis of estradiol [28]. We speculate the estradiol might effect on steroid hormone synthesis by hypothalamus-pituitary-gonad (HPG) axis. On the contrary, high concentrations of steroids might regulate *CYP11A1* mRNA expression.

We also observed higher *CYP11A1* expression in Yangzhou geese than in Zhedong geese during the ovulation period. Yangzhou geese are known for high egg production, while Zhedong geese show low egg production. Yangzhou geese might need to release more pregnenolone to ovulate, so the observed higher *CYP11A1* expression levels may be required to ensure sufficient hormone secretion.

## Conclusions

In summary, we present the molecular cloning and characterization of the goose *CYP11A1*, and analyze its expression during the goose reproductive cycle. The relatively low levels of *CYP11A1* mRNA were detected at ovulation when compared with levels at oviposition. The higher mRNA expression was investigated during the ovulation period of Yangzhou geese than that of Zhedong geese. Our findings provides correlative evidence that *CYP11A1* expression is important in reproduction activity.

## Abbreviations

P450scc: side-chain cleavage cytochrome P450
CYP11A1: cholesterol side chain cleavage enzyme
RT-PCR: reverse transcription polymerase chain reaction
RACE: rapid amplification of cDNA ends
bp: base pair
UTR: untranslated region
qPCR: quantitative polymerase chain reaction
GAPDH: glyceraldehyde-3-phosphate dehydrogenase
HPG: hypothalamus-pituitary-gonad
